# The Nursing Institute

**Published:** 1907-09-21

**Authors:** 


### Nursing Administration.

### THE NURSING INSTITUTE.

Should nursing institutes be under private management? There is no doubt that exceptional women can be named who single-handed have succeeded in building up nursing agencies of high repute. They have catered for the public, the medical men, and the nurses so as to earn the esteem and confidence of all. However, the question is not so much can this thing be done by those who are exceptionally gifted, but ought it to be habitually undertaken by women of ordinary powers? Few people, not actually engaged in the work, have any conception of the difficulties involved in the supply of nurses to the public. It demands in truth talents of a high order if it is to be rightly carried out. The talent for selecting members of the staff, the talent for attracting the support of the medical profession, the talent for pleasing the public, the talent for bringing contentment to nurses working for the institute, these are some of the more apparent gifts which are indispensable to the superintendent. But when these are attained there remains a big series of problems which it is hardly fit that any one woman should be called upon to grapple with alone. The superintendent who is also proprietress is face to face with financial responsibilities which affect not merely herself personally, but also those with whom she is associated. She often calls her undertaking a "Co-operation," but whether she does so or not, it is in fact a co-operation, and her income is built up out of the earnings of others combined with her own. She commonly works harder than any of her staff, but all the same she and the nurses undertake common responsibilities, and the onus of dividing the profits rests on her shoulders.

It is very difficult to ascertain in any given locality what proportion of the fee represents a fair percentage for the agency to receive. If the percentage is set too low a serious loss may easily result to the superintendent in a quiet season, for her expenses will go on as usual and will certainly include centrally situated and expensive premises together with a permanent staff. On the other hand, supposing in a large agency the percentage is even slightly in excess of what may be reckoned a fair profit, the imputation is at once freely launched that the successful superintendent is making a fortune out of the labours of her staff. To steer a true course between Scylla and Charybdis, to be guaranteed against bad debts, and yet give the nurses the full benefit of any prosperity which is the direct result of their good work, is a task which the wisest administrator might fail single-handed to achieve. Practical business ability is needed to forsee liabilities and turn profit to the best account, and since in its initiation the undertaking must pass through a period of unremunerative struggles there should be command of capital to set it on a secure footing. To grapple with these problems and at the same time to conduct the institute to the best advantage for nurses and patients alike is beyond the powers of one woman. It is public work, and as such is best dealt with in association with others. The business difficulties are more adequately dealt with by a small committee of responsible people whose names carry confidence to the public, and the superintendent is unquestionably in a better position to discharge her onerous labours when she is free from financial worry, is guaranteed a good salary whether work be plentiful or not, and can count in any circumstances of difficulty on the support of others. Rare indeed are the instances in which the private agency becomes a really lucrative investment. The receipts may appear to the outsider, calculating merely the percentages received from nurses, to be enormous. But closer acquaintance with the liabilities incurred tends to modify these brilliant appearances. Often there is a nurses' home attached through which money leaks away without affording satisfaction to anyone. There are often bad debts which the superintendent shrinks from the publicity of collecting by legal means. And when nurses are permanently engaged, as in many localities must be the case, there may be loss enough in a bad year to run away with the proceeds of more than one prosperous season. The tendency in private enterprises is to level the rate of living up to the highest rate of profit in any given year, and thus even small depreciations come as calamities.

Now the case for the committee is as follows: In the first place two heads are better than one, and the collective experience of business people tends to caution. The existence of a committee ensures the regular auditing of the accounts, the regular preparation of a balance-sheet, the issue of a report, albeit a private one. It is for want of these precautions that the private institute often gets hopelessly involved. The committee is a court of appeal. It inspires a little wholesome awe in monetary transactions. It is not afraid of suing or being sued, and the mere fact of this being known is sufficient to obviate many minor difficulties whether with the nurse or patient. The committee has the value of permanence. It foresees, builds for the future, regards the passing year as but one in a series and escapes the error of measuring success or failure by temporary vicissitudes. Moreover the committee is free from any personal taint in dealing with the profits of the undertaking. It is concerned to deal liberally with its officers, to accumulate a sufficient reserve for emergencies, and to give to each worker the benefit of her own earnings. Its counsels may not at all times command approval, for absolute wisdom under all conditions is not the property of any group of people however admirable individually, but it is at least under no imputation of self aggrandisement. Its regulations may be open to criticism, but they are made with collective deliberation, and are free from the disastrous effects of personal pique or panic. They are better fitted to meet responsibilities of this particular nature than any private person however highly qualified.

We have regarded the matter from the point of view of the efficient private proprietress of nursing agencies. The public danger and private loss entailed by agencies of a different order, designed purely to squeeze profit from nurses and patients alike, is a serious menace to the nursing profession at large.

